# Home Delivery of Water for Caries Prevention in Latinx Children (“Sediento por una Sonrisa,” Thirsty for a Smile): Protocol for a Single-Arm Feasibility Study

**DOI:** 10.2196/37200

**Published:** 2022-04-15

**Authors:** Joana Cunha-Cruz, Linda K Ko, Lloyd Mancl, Marilynn L Rothen, Catherine Harter, Stephen Davis, Mark Koday

**Affiliations:** 1 Department of Clinical and Community Sciences University of Alabama at Birmingham Birmingham, AL United States; 2 Department of Oral Health Sciences University of Washington Seattle, WA United States; 3 Department of Health Systems and Population Health University of Washington Seattle, WA United States; 4 Department of Assessment, Planning and Development Tacoma Pierce County Health Department Tacoma, WA United States; 5 Dental Department Yakima Valley Farm Workers Clinic Yakima, WA United States

**Keywords:** dental caries, behavioral intervention, environmental restructuring, practice-based research, sugar consumption, feasibility, water consumption, nutrition, oral health, Latino (a) health, dental health, dentistry, dental, public health

## Abstract

**Background:**

Dental caries has significant public health implications afflicting young children. In addition to low social economic status, the most prominent risk factor for early childhood caries is sugar in the diet, particularly sugar-sweetened beverages. Dental treatment for caries in young children is commonly performed under general anesthesia and a significant proportion of children require repeated treatment. Interventions to reduce sugar-sweetened beverage consumption could lead to reduced rates of retreatment for dental caries in young children.

**Objective:**

This protocol describes the rationale, design, and methods of the “Thirsty for a Smile” feasibility study. The aim of the study is to assess the feasibility, acceptability, and appropriateness of a dietary intervention promoting water consumption in lieu of sugar-sweetened beverages among young patients, mostly from Latino heritage.

**Methods:**

This protocol describes a single-arm feasibility study. Twenty-one dyads of children and their caregivers will be recruited. Children between 2 and 9 years old who recently had treatment under general anesthesia for early childhood dental caries will be eligible to participate. The intervention has two components: (1) environmental, in which bottled water is delivered to participants’ homes; and (2) behavioral, in which caregivers will receive patient-centered counseling to increase children’s water intake and reduce sugar-sweetened beverages consumption. Dental caries and anthropometric data will be collected at examination during baseline and final visits. The primary outcome is feasibility and secondary outcomes are acceptability and appropriateness of the intervention.

**Results:**

Funding has been obtained from the National Institute of Dental and Craniofacial Research and the University of Washington approved the study. The feasibility study was conducted from March to November 2019.

**Conclusions:**

This feasibility study will test the study processes prior to a two-arm randomized controlled trial to determine feasibility and acceptability of the intervention and study procedures. This study may provide useful information for other researchers attempting to test similar interventions.

**International Registered Report Identifier (IRRID):**

RR1-10.2196/37200

## Introduction

Early childhood caries (ECC) is a condition with significant public health implications for young children (0-3 years), especially in lower socioeconomic groups [1-5]. In one systematic review, the most prominent risk factor for dental caries was sugar in the diet, in addition to low socioeconomic status (SES) [6]. There is overwhelming evidence that sugar-sweetened beverages (SSBs) are associated with dental caries in young children [7-14]. SSBs—defined as beverages with energy-containing sweeteners such as fruit juice concentrates, sucrose, or high-fructose corn syrup—are the primary source of sugars in the American diet [15]. These drinks represent approximately half of all added sugar consumption of children [16] and children in low SES groups drink more SSBs than those from high-income families [17]. The typical daily consumption of added sugar in 2001 was 12 teaspoons for children 1-3 years old and was 21 teaspoons for children 4-8 years old [18]. For reference, a 12-ounce can of cola contains 8 teaspoons of sugar [19].

The standard of care for dental caries involves surgical removal or restoration of the teeth, application of a topical fluoride, and recommendations to minimize decay-promoting dietary habits such as the frequent consumption of SSBs [20]. Treatment of dental caries under general anesthesia is becoming more common and costly, which has a major impact on families. More importantly, the outcome of this treatment for ECC is poor: 37%-79% of treated children develop new carious lesions within 24 months [21-25] and 17% require surgical retreatment within 2 years [25]. An intervention focused on decreasing dental caries relapse postsurgery would avoid traumatic and expensive dental care.

A comprehensive approach that targets the main causes of the disease is necessary to improve the clinical outcomes for severe dental caries [26]. Unfortunately, the trials of one-on-one dietary counseling undertaken in dental settings have been neither promising nor rigorous [27]. Dietary management of other chronic diseases—specifically childhood obesity—has been successfully implemented in studies [28-30] and can be adapted to clinical settings. Evidence is mounting that interventions to reduce the consumption of SSBs [28,29,31-36], including interventions promoting water consumption to displace SSBs [30], can be effective. Many children with severe ECC (S-ECC) are treated in major academic health centers or hospitals with programs in pediatric dentistry. These centers have sufficient scale to implement new, more effective approaches to the secondary prevention of ECC. Moreover, the lessons learned from preventing recurrent disease can also be applied to primary prevention. Reducing the consumption of SSBs and promoting the consumption of water is an appropriate strategy to prevent dental caries and other chronic diseases.

The primary objective of the proposed study is to test the feasibility of an environmental dietary intervention designed to promote the consumption of water, in lieu of SSBs, on dental caries relapse among Latino and non-Latino children. A secondary objective is to investigate the acceptability of the environmental dietary intervention strategies and tools among families and patients. This short-term feasibility project will refine the study methods and data collection tools to effectively plan and perform a randomized controlled trial (RCT).

## Methods

### Design

This will be a single-arm feasibility study, named “Sediento por una Sonrisa” (“Thirsty for a Smile”). Using a mixed methods approach, we will evaluate the feasibility of the intervention and procedures intended for use in a phase II RCT. Children will be clinically examined at baseline and at a follow-up visit, and caregivers will answer a questionnaire and dietary intake interview.

### Ethics and Confidentiality

The Institutional Review Board (IRB) of the University of Washington (UW) will serve as the IRB of oversight for this study. The protocol, informed consent form(s), recruitment materials, and all subject materials will be submitted to the IRB for review and approval. Approval of both the protocol and the consent form must be obtained before any subject is enrolled. Any amendment to the protocol will require review and approval by the IRB before the changes are implemented in the study.

A consent form describing the detailed study procedures and risks will be given to the potential participant. The investigator or designee will explain the research study to the potential participant and answer any questions that may arise. Study staff conducting the consent process will be bilingual (English and Spanish) to accommodate non-English speakers. All study forms and materials will be available in Spanish and English. Participants will elect their preferred language for all communications. The consent process will be documented in the clinical or research record.

Study participant confidentiality is strictly held in trust by the investigators, study staff, and the sponsor(s) and their agents. This confidentiality is extended to cover any study information relating to subjects. The investigator will ensure that this study is conducted in full conformity with the principles set forth in The Belmont Report: Ethical Principles and Guidelines for the Protection of Human Subjects of Research, as drafted by the US National Commission for the Protection of Human Subjects of Biomedical and Behavioral Research (April 18, 1979) and codified in the US Code of Federal Regulations Title 45: Public Welfare, part 46 and/or the International Council for Harmonisation’s Guidelines for Good Clinical Practice E6.

This project does not meet the National Institutes of Health (NIH) definition of a clinical trial as it will not study the cause-and-effect relationship between an intervention and a health outcome. Therefore, this study will not be registered in a public trials registry such as ClinicalTrials.gov.

### Participants and Setting

Inclusion criteria for parents/caregivers and child dyads for enrollment include providing a signed and dated consent form, willingness to comply with all study procedures, availability for the duration of the study, a child aged 2 to less than 9 years, a diagnosis of S-ECC, previous treatment for ECC under general anesthesia, and a child in good general health based on parent self-report. The study clinical site treats approximately 700 pediatric patients with ECC per year; approximately 500 of these patients would be eligible to participate in the study with a goal of enrolling 21 participants. Exclusion criteria for parents/caregivers and child dyads include a parent/caregiver under 18 years of age or a child with American Society of Anesthesiologists Physical Status IV or higher.

Caregivers will be recruited by the onsite research coordinator during the initial examination visit at the dental clinical site once the child is determined to have S-ECC and to meet the other eligibility requirements. A promotional pamphlet or flyer will be distributed to caregivers of children scheduled to undergo surgical treatment for S-ECC by the research coordinator.

### Study Intervention

#### Overview and Aims

**Figure 1:**
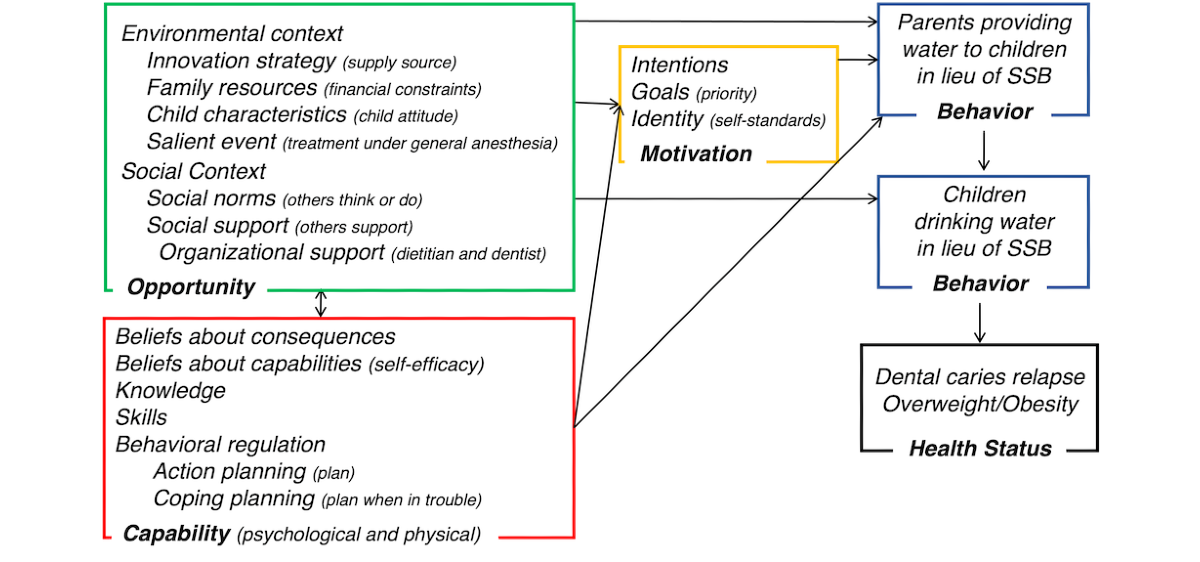
Conceptual framework using the Capabilities, Opportunities and Motivation-Behavior (COM-B) and Theoretical Domains frameworks. SSB: sugar-sweetened beverage.

The conceptual model of the study proposes that families have a high degree of control over children’s environments, with parents being the most influential in the selection of diet [37] and the development of eating and drinking behaviors [38,39]. Family practices associated with beverage choices include the availability and accessibility of water and SSBs in the home environment [40-43], and role-modeling beverage choices [41,44-47] and habits such as drinking water between meals [48]. Lack of knowledge, beliefs, and skills such as self-efficacy, action planning, and motivation, along with social norms, support, and environmental opportunities are intra- and interpersonal barriers for healthy drinking and eating [49-51], which can in turn increase the risk of dental caries [52] and obesity [53,54] (Figure 1).

The intervention aims to provide parents with physical and social opportunities, motivation, and physical and psychological capabilities (knowledge and skills) to modify three key domains within the home environment. The first domain is the availability and accessibility of water: the intervention encourages parents to ensure that water is available and accessible in the home, and that it is prepared, presented, or maintained in a ready-to-use form that encourages consumption. The second is supportive family eating routines: the intervention will seek to improve parent knowledge and facilitate the acquisition of skills to support the children to drink water and milk at meals and water at all other times, establish and enforce family rules about drinking, and develop boundaries regarding when and how SSBs are offered to the children. The third domain involves parental role-modeling of water consumption: parents will be encouraged to increase the number of servings of water that they consume in front of their children and to express supportive attitudes toward the consumption of water to their children, such as by making positive and encouraging comments. The two components of the intervention are: (1) a dietary-environmental change, which involves home delivery of child-friendly bottles of fluoridated water to replace SSB choices; and (2) a behavioral counseling intervention intended to increase parents’ knowledge of the adverse effects of SSB consumption on young children’s oral health, along with the strategies and skills needed to reduce SSB consumption.

#### Product Description

The product is fluoridated water packaged in child-friendly, brightly colored, animal-shaped plastic bottles with resealable spouts. The product will be obtained from a specialty drink manufacturer in Mukilteo, Washington. Specifically, the water will be packaged in individually sealed 8-ounce food-grade plastic containers, and the boxes will be labeled with the study name, contents, and information about how to contact the investigators. Bottles will be shipped by a commercial carrier from the manufacturer to the clinical site storage and then delivered to the enrolled subjects. Included are instructions to consume up to 3 bottles per day as the water contains approximately 0.7 parts per million fluoride.

#### Behavioral Intervention: In-Person Counseling

The behavioral component will provide a combination of in-person and telephone counseling by trained dietitians to help parents overcome barriers to decreasing their children’s consumption of SSBs and increasing their consumption of water. By involving parents and children, the intervention has the potential to establish new family norms for beverage consumption.

The first in-person session will be approximately 30-45 minutes in duration. Following the first in-person session, parents will receive four telephone check-in visits. The calls will be weekly for 3 weeks after the first in-person visit and a fourth call will occur at the middle of the second month. Telephone interventions have become increasingly popular for health education and behavior change counseling, as they are convenient and cost-effective [55]. There will be one final in-person session of approximately 20 minutes duration at the final visit at 2 months. Sample scripts for the three types of counseling sessions will be provided to the dietitians.

#### Procedures for Training Interventionists and Monitoring Intervention Fidelity

Training of the dietitians delivering the counseling intervention occurs in person and will follow a manual written for this study. Interventionists will be trained to defined performance criteria and be “certified.” During the training, interventionists will be asked to undertake role-playing exercises, which will be scored according to preestablished checklists of the essential elements necessary in each type of counseling session (eg, assessing motivation, goal-setting, and anticipating challenges). After the role-playing exercises, interventionists will evaluate their own performance and identify, along with the trainer and other participants, aspects that would benefit from additional practice or improvement. This additional work will be completed during the training days or by phone immediately following the training.

Fidelity monitoring will be guided by recommendations of the Treatment Fidelity Workgroup of the NIH Behavior Change Consortium [56]. To assess fidelity, we will audio-record approximately 25% of each type of session (first and final in-person visits, telephone check-ins) delivered by each interventionist and provide individualized feedback. The trainer will listen to the audio recordings and evaluate fidelity using the training checklists designed for each type of counseling session. Intervention fidelity ensures that the intervention was conducted as planned (ie, follows the study protocol).

The onsite research coordinator will oversee the activities of the clinical site and will report to the project principal investigators. Staff training for the research coordinator, clinic dietitians, and dental hygienists will occur onsite at the clinical site. Although the training varies depending on the staff member’s role in the project, at minimum, all staff will be trained in the topics of Human Subject Protection, Good Clinical Practice (GCP), and protocol training.

### Procedures

#### Study Schedule

##### Screening, Recruitment, and Enrollment

Caregivers will be recruited during the initial examination visit at the dental clinic once the child is determined to meet eligibility requirements. The dental provider will introduce the study and a study coordinator will continue the recruitment and consent processes.

The process will include reviewing the written consent form with the potential participant; the study staff signs the consent form acknowledging that informed consent was reviewed and the caregiver signs acknowledging that consent was obtained. We will also obtain demographic and contact information as well as the record time for recruitment and consent.

##### Baseline and Final Visit

The examiner will conduct a dental caries exam, and measurements of height, weight, and waist circumference (anthropometrics) will be obtained from the child and their primary parent/caregiver. Time for each data collection element will be recorded. Caregiver/parent participants will complete the Child Oral Health Questionnaire where time to completion will be recorded. The caregiver/parent participants will then participate in an in-person counseling session with the clinic dietitian where time will be recorded.

Phone interviews will be conducted with caregiver/parent participants at baseline and at the final visit at 2 months to collect 24-hour dietary recall data. Interviews will last approximately 45 minutes and the time will be recorded.

##### Intermediate Phone Call Follow-ups

The clinic dietitian will call participants weekly for the first month and then once at the middle of the second month to check in about behavior change progress and address any questions or concerns regarding the topics covered during the in-person counseling sessions. The amount of time per check-in call will be recorded and the number of attempted phone calls will be tracked.

#### Data Collection

Data for this study will include: (1) dental examination data, (2) height and weight measurements, (3) study questionnaires to assess oral health–related quality of life and dietary behavior, and (4) dental claims data. Dental hygienists will be trained to collect the dental caries measurements using the International Caries Detection and Assessment System. Data for this study are captured using examinations, standardized forms, and questionnaires. The investigators will maintain adequate case histories of study subjects, including accurate case report forms and source documentation.

Data will be entered directly into the Research Electronic Data Capture (REDCap) system, including the results of the physical examinations, questionnaires, and intervention counseling notes; time and staff conducting the activities; and reasons for unsuccessful attempts and actions to overcome barriers.

Paper forms will be securely stored in a locked file cabinet when they are not in use. Completed paper forms will be transferred between sites via courier or another secure document service. Dietary recall data will be entered directly into the secure web-based system and data will be securely transferred to UW. Dental claims data will be securely transferred to UW.

#### Assessment of Safety

Safety parameters for this study include monitoring unanticipated problems, adverse events, serious adverse events, and time and frequency for event assessment and follow-up.

Incidents or events that meet the US Office for Human Research Protections (OHRP) criteria for unanticipated problems require the creation and completion of an unanticipated problem report form. To satisfy the requirement for prompt reporting, unanticipated problems will be reported using the following timeline: unanticipated problems that are serious adverse events will be reported to the UW IRB and to the sponsor within 1 week of the investigator becoming aware of the event; any other unanticipated problem will be reported to the UW IRB and to the sponsor within 2 weeks of the investigator becoming aware of the problem. All unanticipated problems should be reported to appropriate institutional officials (as required by an institution’s written reporting procedures), the supporting agency head (or designee), and the OHRP within 1 month of the IRB’s receipt of the report of the problem from the investigator. All unanticipated problems will be reported to the sponsor’s centralized reporting system.

#### Quality Control

A sample of study staff telephone contacts (reminder calls for study and follow-up appointments) and of each type of the 3 counseling sessions (first in-person, last in-person, and telephone check-ins) will be audio-recorded in digital media and reviewed (approximately 25% of the study staff–caregiver intervention interactions). The study investigator will then review the telephone contacts to ensure fidelity of the calls and accuracy of data recording. The staff will be provided with feedback on their performance and conduct, including but not limited to these specific areas: adherence to the telephone script, adherence to the protocol/manual of procedures, and adherence to GCP. Clinic staff with a telephone contact indicative of less than acceptable quality will receive a private training session focused on problematic areas. The dietitians will have their audio recordings reviewed by a behavioral psychologist and will be provided with feedback.

### Measures

#### Primary Objectives

To assess feasibility of the intervention, we will determine if study procedures and processes can be executed and the resources and time necessary for execution. We will track how much and what type of support are needed to deliver the intervention, such as home delivery of water bottles, and how much staff time and resources it took to implement the study. To assess execution of study procedures and processes, we will collect the following information (goals in parentheses): the participation rate among eligible caregivers and children (≥50%), the follow-up rate at the end of the 2-month study period (≥90%), the number of water bottles delivered and on time (≥90%), compliance with the recommended water consumption based on the delivered water bottles, the number of completed behavioral intervention sessions (100% at baseline, ≥90% for weekly sessions, and ≥90% at the final follow-up), reasons for noncompletion of intervention sessions, intervention fidelity, the number of completed dietary recall assessments at baseline (100%) and at the final follow-up (≥90%), and the number of dental and physical exams completed at baseline (100%) and final follow-up (≥90%).

#### Secondary Objectives

We will assess the acceptability, appropriateness, and perceived feasibility for each of the intervention components and health assessments, and the willingness to participate in a longer trial (1-year intervention plus 1 additional year of follow-up) (≥80%).

The questions to assess the intervention components were developed by the investigators and participants will be asked to rate each component on a scale from 1 to 10. Acceptability of survey completion will be assessed using questions adapted from Wolpin et al [57], which have been used to assess acceptability of an electronic self-report program for cancer patients. The six Likert-scale items, measured from 1 (“not at all”) to 5 (“very much”), ask about ease of use, satisfaction, helpfulness, and completion time.

### Statistical Considerations

#### Power

The sample size is primarily based on what is feasible within the constraints of the funding mechanism with respect to cost and length of the grant period. The proposed sample size is 21 caregivers and children, which will provide estimates with sufficient precision for sample size determination for the secondary outcomes in the trial. Estimates for quantitative outcomes will have a precision of ±0.30 SD and estimates for subjective outcomes will have a precision of ±10%, based on an 80% CI. Estimates for the primary outcome (caries relapse after 2 years) will be obtained from other sources.

#### Statistical Analysis

The data analysis will be descriptive, using frequency and percent for categorical measures and mean (SD and range) for quantitative outcomes. No a priori hypotheses will be tested. The 80% CIs will be computed to describe the precision of the estimates for both aims and to guide sample size determination for the larger study.

The qualitative analysis will be based on data from a focus group comprised of caregivers of child participants involved in the study and interviews as needed for those who cannot attend the focus group. The interviews will be conducted and recorded in Spanish, transcribed, and translated into English and checked for accuracy. Coding will be completed by two researchers, first individually and then they will meet to discuss and agree upon codes. Researchers will utilize an inductive process to thematically analyze codes.

## Results

Funding has been obtained from the National Institute of Dental and Craniofacial Research (NIDCR) and the UW IRB has approved the study. The feasibility study was conducted from March until November 2019. Data analysis is currently underway and the results are expected to be published by the end of 2022.

## Discussion

### Hypothesis and Significance

We hypothesize that a combined dietary environmental and counseling intervention—home delivery of bottled water and parental counseling regarding water consumption—will lengthen the time to onset of new dental caries relative to the current standard of care for pediatric patients with S-ECC. The intervention to be evaluated in this study is family-focused [58] and introduces new familial norms by making changes in the home environment (more attractive water choices, fewer SSBs, and parental role-modeling of a preference for water over SSBs). It is expected to strengthen caregiver self-efficacy for implementing change in the child’s diet. Parents assume a leadership role in implementing the intervention and in negotiating buy-in from other adults and older children present in the home.

The main behavioral change techniques used will be physical environmental restructuring by adding an object to the environment (water bottles with fluoridated water delivered twice a month to the home environment for 2 months). Water bottles are being used because they are acceptable to the families and because many of the potential participating families—who have immigrated to the United States—distrust tap water safety [59-61].

The physical environmental restructuring will be supported by providing a service (behavioral counseling) to restructure the social environment of the child using self-regulatory techniques. Behavioral counseling to caregivers about healthy drinking for their children will change parental skills and self-efficacy on the child’s healthy drinking. Consumer Information Processing Theory [62] posits that health information must be wanted, understood, and presented at a time the patient is most receptive. Thus, we will initiate the behavioral counseling at the child’s first recall visit to the dental clinic following surgery, typically 14-21 days postdischarge, when parents and children are no longer preoccupied by the upcoming surgery but are in an early stage in their transition to reestablish home routines.

The in-person and telephone check-in sessions consist of motivational techniques [63] to increase the parents’ self-efficacy [64] about their ability to increase their child’s water drinking and decrease their SSB consumption. These sessions include volitional techniques to facilitate realistic goal-setting [65] and to translate intentions into routine practices (eg, action and plans) [66]. Throughout, parents are encouraged in “cope planning,” to identify factors that support or hamper behavior change, and make plans to bring about the supportive factors and overcome the barriers [66]. The final in-person session focuses on plans to maintain behavior changes in the absence of the home water delivery and a discussion of relapse prevention.

### Limitations

The sample size is relatively small and the length of time for the intervention is only 2 months. As noted, this is a feasibility study and larger studies will be needed to provide adequate power and assess the effectiveness of the intervention in decreasing S-ECC. Participation may be hindered by the nature of human behavior and the time constraints of families with multiple children with competing needs. Because our focus here is on ECC, we have a limited age range; however, children older than 8 years may also benefit from this dietary-environmental intervention. The feasibility and acceptance outcomes may not be generalizable and may be specific to the community and setting. However, the feasibility study will allow the investigators and the clinical site staff to test recruitment and retainment of participants, intervention delivery, and outcome assessments at the clinical site.

### Conclusions

Expected outcomes of this study will provide a basis for a successful RCT. Results of this project will produce an intervention suited to the environment and people who will use it, and will indicate challenges and opportunities for refinement of the intervention and study procedures, including how much and what type of support are needed, and how much staff time and resources it will take to implement the study.

## Appendix

Multimedia Appendix 1 National Institute of Dental and Craniofacial Research (NIDCR) Peer Review Report.

## Abbreviations

ECC: early childhood caries
GCP: Good Clinical Practice
IRB: Institutional Review Board
NIDCR: National Institute of Dental and Craniofacial Research
NIH: National Institutes of Health
OHRP: Office for Human Research Protections
RCT: randomized controlled trial
REDCap: Research Electronic Data Capture
S-ECC: severe early childhood caries
SES: socioeconomic status
SSB: sugar-sweetened beverage
UW: University of Washington
