# Supplementary material for: Home Delivery of Water for Caries Prevention in Latinx Children (“Sediento por una Sonrisa,” Thirsty for a Smile): Protocol for a Single-Arm Feasibility Study
Source: JMIR Res Protoc. 2022 Apr 15;11(4):e37200. doi: 10.2196/37200 (PMC9055491; doi:10.2196/37200)
Supplement: Multimedia Appendix 1 [file resprot_v11i4e37200_app1.pdf]

**1U01DE027026-01A1 CUNHA-CRUZ, JOANA**

**EARLY STAGE INVESTIGATOR**
**NEW INVESTIGATOR**

**RESUME AND SUMMARY OF DISCUSSION:** This resubmission application is submitted by the University of Washington on behalf of Joana Cunha-Cruz, DDS, PhD, in response to the NIDCR Clinical Trial or Biomarker Clinical Validation Study Cooperative Agreement (U01), PAR-15-059. The goal of the research is to test an intervention aimed to slow the relapse rate of early childhood caries (ECC) after surgical care, allowing time for the child to develop secondary dentition and learn positive oral health and nutritional behaviors. The research will utilize a randomized phase II clinical trial with a cohort of 260 children who have been surgically treated for ECC, to study the efficacy of replacing sugar sweetened beverages (by providing home delivery of water bottles) combined with family-based parental counseling on relapse of ECC, weight gain, and sugar sweetened beverage consumption, as compared to current standard of care (parent health nutrition education). The primary outcome to be determined is the time to onset of new carious lesions over two years and two secondary outcomes to be measured are body mass index for age (BMI-for-age) and sugar sweetened beverage consumption. The main strengths of this application are the exceptionally high significance of reducing the recurrence of ECC in children and improving both the oral and nutritional health of children and their families. Further significance is noted in the potential economic impact resulting from the reduction of related health care costs. Using the measure of time to caries relapse as the primary outcome, is novel and may provide better results and more useful data than a clinical outcome measure; and including the collateral measures of obesity is a thoughtful addition to the study framework. The study design is robust and the scientific premise is well supported. The dedicated Principal Investigator (PI) and gathered research team have appropriate expertise and active knowledge of the cohort to be studied. The study documentation is thorough and the research and public health environment is exceptional. The resubmission is rated as acceptable. A few minor weaknesses are noted. The study design does not adequately consider that the retention of subjects may be influenced by the same social impacts causing caries relapse. The study also does not clearly control for the concomitant use of additional dental care that may be available to the subjects. There is no consideration for the possibility of intervention envy in the potentially small communities; with control families seeing the delivery of water to neighbors. There was disagreement on the panel regarding the long-term sustainability of the intervention: some reviewers see the intervention to be effort and cost intensive, while other reviewers noted that, if effective, the intervention would be much less costly than the oral health care costs associated with ECC. Finally, there is concern that the travel burden on families for the intervention and data collection is high, potentially impacting measures such as water consumption tracking through empty water bottle return. The Protections of Human Subjects and Clinical Trials documentation are rated as acceptable. The potential impact of this proposed phase II clinical trial, to study the efficacy of reducing ECC recurrence and obesity through an intervention replacing sugar sweetened beverages with water, is deemed very high.

**DESCRIPTION (provided by applicant):** Sugar sweetened beverages (SSBs) are a common risk factor for tooth decay Early Childhood Caries (ECC), and obesity. Both are serious problems; particularly for Latino children, as they are more likely to drink SSBs and less likely to drink water. Our primary aim is to test the efficacy of home delivery of water bottles with family-based parental counseling on relapse of ECC, weight gain and SSB consumption compared to usual care (parent health nutrition education). Our secondary aim is to investigate the mechanism of action of the intervention on sugar consumption, caries relapse and weight gain through analysis of mediators, moderators and confounders. Our rationale is that families lack the capability (skills, self-efficacy) and opportunities (child-friendly water bottles, social support), but are motivated to reduce SSB

consumption, particularly after dental treatment under general anethesia. Our oral health goal is to slow the rate of relapse of ECC so that teeth can exfoliate normally or allow for time for the child to develop coping skills for less-costly in- office dental treatments without sedation. ECC impacts quality of life, causes pain, eating and sleeping problems. The care standard of surgical care under general anesthesia fails to address the cause of ECC, and the disease frequently relapses. In addition, hospitalization and general anesthesia divert scarce treatment resources away from public health efforts to prevent the ravages of the disease. Drinking water instead of SSBs can reduce children's total daily sugar intake thereby reduce both ECC and obesity. METHODS: 260 children (age < 9 years, 50% Latino) who had treatment for ECC under general anesthesia and their families will be randomly assigned to the experimental intervention or usual care (nutrition health education). Participants in the intervention will have fluoridated water in cute brightly-colored animal- shaped bottles delivered bi-weekly for one year to their homes coupled with family-directed, monthly caregiver counseling provided by bilingual dietitians to support implementing change in beverage consumption. The intervention employs established behavioral change techniques shown to impact dietary change in young children: environmental and social opportunities and caregivers' stimulus control, prompt identification as role model and self-regulation. The primary outcome is time to onset of new dental caries lesions through two years. Secondary outcomes are body mass index for age (BMI-for-age) and SSB consumption. SIGNIFICANCE: This study will provide the best evidence to date of whether changing the home environment and empowering families can promote water intake, in lieu of sugar sweetened beverages, to reduce ECC and obesity in children. We address gaps in knowledge in nutrition in the home and theory on means to change dietary habits. The proposal for research is a unique multidisciplinary collaboration between University of Washington and the Yakima Valley Farm Workers Clinic serving immigrant Latino families. The results will serve as a step toward trials to generalize findings and scale-up effective interventions.

**PUBLIC HEALTH RELEVANCE:** This is a five-year application from the University of Washington and Yakima Valley Farm Workers Clinic to support a Phase II randomized clinical trial to assess the efficacy of an environmental dietary intervention, among Latino children with severe early childhood caries to increase time to relapse of dental caries and reduce obesity. This trial addresses Healthy People 2020 Objectives OH-1 to reduce the proportion of children who have dental caries experience in their primary or permanent teeth and NWS-10.4 to reduce the proportion of children and adolescents with obesity.

**CRITIQUE 1**

| | |
|---|---|
| Significance: | 2 |
| Investigator(s): | 1 |
| Innovation: | 1 |
| Approach: | 3 |
| Environment: | 1 |

**Overall Impact:** This is a resubmission application to conduct a randomized controlled trial in young children testing the ability of displacement of sugar-sweetened beverages and educational intervention to reduce time to relapse in children who have received surgery for ECC. The target population is recruited from a clinic network in Yakima Valley, WA, which mainly serves low-income Latino immigrant families. In this resubmission, significance remains high. The intervention fills an important gap and targets a high-risk vulnerable population. The application is innovative in its approach to target both obesity and oral health outcomes, and in its choice of a primary outcome. The investigators and environment are excellent with no weaknesses noted. The study design is strong. The intervention has a good evidence base. References are provided for several studies that describe successful displacement of sugar-sweetened beverages with water provision in adolescents and adults. While these are different populations, they imply the intervention should work and are appropriate evidence to support a Phase II trial. Some challenges remain. The main intervention ends at 12-months but the

primary outcome is at 24-months which seems an overly pragmatic approach for a phase II trial. The study includes children up until age nine even though it is focused on caries relapse in preschool children. It is still unclear how families will share the water bottles and the burden on families to travel for intervention and data collection is high. Despite these issues, the application is strong overall. Changing the timing of the primary outcome affects power and reducing the eligible age affects recruitment, but both seem to be issues that could be resolved in a cooperative agreement.

**1. Significance:**
Strengths
- Targeting children who already have had severe caries with an aggressive dietary intervention such as this is appropriate and important. Well-tested dietary interventions to prevent relapse of ECC are lacking.
- Existing evidence support the proposed approach to target the home environment, providing education but also tangible resources (water bottles) to support families to make changes.
- Low income predominantly Latino children are one of the highest risk groups and therefore the right population for this intervention.
- Inclusion of children under the age of five years old attempts to instill healthy behaviors for a lifetime, before the recurrence of caries.
- Measuring obesity outcomes along with oral health is a great use of resources and helps enforce to families the multiple potential benefits of the intervention.

Weaknesses
- The sustainability of the intervention as proposed (delivery of large amounts of free water bottles twice a month) is questionable due to the expense and operational challenges. While public health systems do provide water in some areas, it is because public water is unsafe. This also raises questions of environmental responsibility (plastic production and disposal, fuel for transportation). If successful, future iterations of the intervention will need to address this.

**2. Investigator(s):**
Strengths
- The investigative team is strong and well-suited to conduct the proposed research.
- The Principal Investigator (PI) is a dentist and epidemiologist who has worked with the proposed team and study partners. She has experience with community-based participatory research and oral health disparities.
- The rest of the team includes senior-level oral health researchers, nutrition researchers, a specialist to oversee collection of the dietary data, a health psychologist, and a biostatistician.
- The Child Dental Officer of the partner clinic is included as a co-investigator.

Weaknesses
- None noted.

**3. Innovation:**
Strengths
- Inclusion of obesity is innovative.
- The study uses a rigorous design to test if water actually can displace sugar-sweetened beverage consumption, and then tests if this dietary change modifies caries outcomes.
- The selection of time to relapse is an innovation in the field.

Weaknesses
- None noted

**4. Approach:**
Strengths
- Single-blind parallel group randomized control trial (RCT) with primary outcome of time to relapse is an appropriate study design. The comparison group is not an attention control and the

intervention is weak in that group. This is good for determination of intervention effect, although it could be viewed negatively by the community. However, the community partners seem actively involved in the study proposal so the assumption is they are in agreement with this plan.
- The animal-shaped bottles are age appropriate, vetted with the target population, and sufficient quantity to meet most of the enrolled children's water needs.
- Motivational interviewing is used for the education.
- Water consumption is partially determined by collection of used bottles. This is a good objective measure to determine consumption, and will serve as a marker for problems (such as families not using the water, selling the bottles or giving them away).
- The process for measuring caries is strong. Measuring food and beverage consumption is difficult; the Food Frequency Questionnaire is one of the best ways to date and the Fred Hutchinson team has experience with this.
- The power and analysis plan appears well thought out.

Weaknesses
- The primary outcome is placed after a "maintenance" period of no intervention delivery for one year. This is a very pragmatic approach. For a phase II study, it might make more sense to have the primary outcome at 12-months to know if the intervention improved outcomes or not. The question of if behaviors are sustained could be a secondary aim.
- It is unclear why the eligible age range extends to age nine. The proposal refers throughout to preschool-age children, but preschool ends at five years old. By five, children spend more time at school, make more independent decisions, and are developing permanent teeth.
- Water consumption is partially determined by collection of used bottles. However, it may not be easy for families to transport the bottles and this cannot be assessed if families do not come.
- The study indicates extra bottles will be given to family members but does not specify how many. Multiple children will want water bottles. Also, it is not clear if the water needs of adults are met other than through education.
- Five in-person sessions to discuss water consumption was mentioned in the initial review as perhaps more than families would need (they would run out of things to discuss). The resubmission includes details on behavior change planning which is good and does take time. However, the travel required by families, especially in rural communities, might make adherence to the intervention challenging for a 20-minute behavioral change discussion. Adherence might be improved if sessions were allowed to be longer and targeted more dietary issues. Or if sessions were timed with planned dental follow-ups.
- The reimbursement plan for data collection (in-person and via telephone) is unclear.
- (minor) The Food Frequency Questionnaire can be difficult to administer in some immigrant families. It is not clear whether the investigators have experience with this type of data collection in the target population.

**5. Environment:**

Strengths
- The majority of the team is out of the University of Washington. The School of Dentistry, School of Public Health, and CTSA are all involved.
- The Yakima Valley Farm Workers Clinic has 26 outpatient sites, a dental residency, their own community advisory board (although a new one will be convened for this study), and is a great partner for this type of research.
- Fred Hutchinson Cancer Research Center will conduct the dietary recalls and analyses of nutrition data.

Weaknesses
- The letters of support are from 2016.

**Clinical Trial Documentation:** The study protocol and MOP are adequate.

**Plans for Patient Recruitment/Retention:**

- The recruitment plan is appropriate. Clinic records will be reviewed, potentially eligible families approached before surgery and then confirmed enrollment after surgery. Passive enrollment will also be used.
- It would be helpful to see the exact numbers of surgeries performed per month at the target clinics for the specified age group to be confident in the recruitment timeline.

**Safety Monitoring:** Adequate

**Protections for Human Subjects:** Acceptable Risks and/or Adequate Protections

- Adequate

Data and Safety Monitoring Plan: Acceptable

- Adequate

**Inclusion of Women, Minorities and Children:**

- Sex/Gender: Distribution justified scientifically
- Race/Ethnicity: Distribution justified scientifically
- For NIH-Defined Phase III trials, Plans for valid design and analysis: Not applicable
- Inclusion/Exclusion of Children under 18: Including ages <18; justified scientifically
- Adequate

**Vertebrate Animals:** Not Applicable (No Vertebrate Animals)

**Biohazards:** Not Applicable (No Biohazards)

**Resubmission:** In the prior review, this application was felt to have high significance and innovation. The investigators were strong and the environment excellent. The overall design and methods were appropriate. Concerns were raised about the acceptance of the rest of the household to the intervention, the strength of preliminary data supporting the intervention, retention, power, and the potential for fluoride varnish to mask intervention effects. Issues raised in the prior review have all been addressed. More details about household acceptability, preliminary data, retention, and power are added. Fluoride varnish is not mentioned, so it is assumed to have been removed from the intervention which seems appropriate.

**Resource Sharing Plans:** Acceptable

**Authentication of Key Biological and/or Chemical Resources:** Not Applicable (No Relevant Resources)

**Budget and Period of Support:** Recommended budget modifications or possible overlap identified:

- If 130 families receive 60 bottles every two weeks for a year: 26x60x130=202,800. The letter from the manufacturer says the bottles are $1 each. So, the total cost is $202,800 not including shipping and ultimate disposal. It is unclear if this is fully budgeted.

**CRITIQUE 2**

| | |
|---|---|
| Significance: | 1 |
| Investigator(s): | 1 |
| Innovation: | 1 |
| Approach: | 2 |

Environment: 1

**Overall Impact:** Closely adhering to established behavioral theory and understanding of caries pathogenesis, this well-designed study seeks to determine whether providing families of young children affected by ECC with child-friendly water bottles to substitute for sugar sweetened beverages can delay or prevent caries progression post-operatively over one to two years compared to children not receiving the water bottles. This study also addresses weight gain in young children as sweetened beverage consumption is a common risk factor for both conditions. This resubmission substantially addresses prior critiques. It is supported by a complete and detailed Manual of Procedures developed under an R34 grant to guide a strong investigator team in an appropriate environment. The application appropriately addresses a range of vagaries inherent in behavioral studies. The investigators are appropriately prepared to conduct the proposed trial.

**1. Significance:**
Strengths
- The proposed behavioral trial is significant as it addresses two highly important, prevalent and consequential chronic conditions of early childhood: early childhood caries (ECC), and obesity. With regards to ECC, it is timely, needed, holds strong potential for scalability and dissemination and is not duplicative. The research will contribute to the science of behavioral caries management. The scientific premise is clear, it addresses a recognized gap in knowledge about how to effectively promote a specific behavior in a high-risk population, including putative mechanisms of action.
- The rationale for selecting the proposed intervention is clear as frequent ingestion of sugar sweetened beverages is considered a primary risk factor for ECC.

Weaknesses
- none

**2. Investigator(s):**
Strengths
- The proposed team is strong, reflecting the full range of expertise needed to conduct the proposed study.

Weaknesses
- None

**3. Innovation:**
Strengths
- The application shifts practice paradigms by engaging in inter-professional care that addresses two conditions through a common pathway.
- The clinical dental team is expanded to include nutritionists.
- The intervention is person- and family-centered and home-based.
- The study addresses the significant but under-attended problem of ECC progression after surgical repair.
- The research will contribute to understanding behavior beyond the particular study population.

Weaknesses
- None

**4. Approach:**
Strengths
- Overall: Study is well grounded in behavioral theory and a conceptual model. The setting, target population, and recruitment strategy are appropriate. Stakeholder engagement is evident.

- MOP: The Manual of Procedures is complete and detailed. Through specific procedural instructions, it addresses and seeks to control for the many vagaries inherent in community-based behavioral studies.
- Controlling for other ECC risk factors: The application is strong in identifying, addressing, and analyzing the range of likely confounders as they seek to isolate the role of beverage consumption in a multifactorial disease.

Weaknesses

- Randomization and contamination: Randomizing after families are recruited from two sites raises question of whether families from either clinic who are assigned to different arms of the study may know each other and be familiar with the difference in study arms leading to possible contamination (e.g. sharing of water bottles across arms).
- Controlling for concomitant dental treatment: The existing clinic policy is to follow children for dental treatment every three months postoperatively presumably to provide preventive treatments as recommended by AAPD which include fluoride applications. For those who are compliant, such frequent applications may overwhelm the impact of sweetened beverage substitution by water suggesting that the sample size may need to be larger to accommodate for this confounder.
- Retention and adherence: The study population confronts multiple constraints and competing priorities that suggest that those who elect to participate and those who adhere to the protocol may be qualitatively different from those who do not – resulting in intervention and control arms populated with more motivated and compliant families. While the research plan and MOP well address recruitment and adherence protocols, it may be additionally helpful to compare caries progression in both arms with caries progression in children who are not enrolled in the study through a retrospective chart review from the dental clinics.

**5. Environment:**
Strengths

- The environment contributes to the likelihood of success.

Weaknesses

- None noted

**Clinical Trial Documentation:** The trial documentation is complete and thorough.

**Plans for Patient Recruitment/Retention:** Plans for patient recruitment and retention are thoroughly described in the application and MOP.

**Safety Monitoring:** Safety monitoring is addressed in the MOP.

**Protections for Human Subjects:** Acceptable Risks and/or Adequate Protections

- This study presents minimal risks to children and parents. The application's description of human subject’s protections is thorough and appropriate.

Data and Safety Monitoring Plan: Acceptable

- The DSMP is thorough and compliant with all requirements of an NIH-sponsored clinical trial.

**Inclusion of Women, Minorities and Children:**

- Sex/Gender: Distribution justified scientifically
- Race/Ethnicity: Distribution justified scientifically
- For NIH-Defined Phase III trials, Plans for valid design and analysis: Not applicable
- Inclusion/Exclusion of Children under 18: Including ages <18; justified scientifically
- Inclusion of children under age 9 as proposed is scientifically justifiable as the study addressed caries and weight gain in young children - two public health problems that are specific to young children.

**Vertebrate Animals:** Not Applicable (No Vertebrate Animals)

**Biohazards:** Not Applicable (No Biohazards)

**Resubmission:** The resubmission addresses prior critiques

**Resource Sharing Plans:** Acceptable

- The resource sharing plan supports open and timely dissemination or research materials and findings.

**Authentication of Key Biological and/or Chemical Resources:** Not Applicable (No Relevant Resources)

**Budget and Period of Support:** Recommend as Requested

**CRITIQUE 3**

Significance: 2
Investigator(s): 1
Innovation: 2
Approach: 3
Environment: 3

**Overall Impact:** In this resubmission application, investigators plan to randomize 260 predominately Latino children with a history of early childhood tooth decay into two arms: 1) an intervention arm that receives biweekly shipments of fluoridated water in cute brightly-colored animal shaped bottles for one year to their homes coupled with family-directed, monthly caregiver counseling provided by bilingual dietitians to support implementing change in beverage consumption; or 2) a usual care arm (nutrition health education). Primary outcome is time to relapse of early childhood tooth decay in the two-year period after randomization. The application comes from a team of experienced investigators. Overall, the grant is strong; however, several considerations with regards to the approach may improve the impact of the work.

**1. Significance:**
Strengths

- This study addresses a common risk factor (sugary sweetened beverages) for dental caries and obesity.
- The focus on home-based consumption of sugary beverages is a strength, given a large proportion of consumption is at home and few studies have addressed home-based approaches for children.

Weaknesses

- None noted.

**2. Investigator(s):**
Strengths

- The investigators have a track record of productive collaborations and bring relevant areas of expertise in dental caries, Latino health, clinical trials and behavioral interventions to the project.

Weaknesses

- None noted.

**3. Innovation:**

Strengths

- Studies addressing sugary beverages, obesity, and dental carries in Latinos are sparse; this RCT addresses the gap in knowledge.

Weaknesses

- Several currently funded studies are addressing family-based interventions to prevent obesity.

**4. Approach:**
Strengths

- The intervention protocols and measures draw heavily from those found to be appropriate in previous studies by the investigators.
- Well thought-out fidelity plan.

Weaknesses

- It is unclear why there is not just a focus on Hispanics seen at the Yakima Valley Farm Workers Clinic. This would allow tailoring to Hispanics and tailoring to the non-Hispanics that attend. Otherwise, there is insufficient detail about how tailoring to each group will be accomplished beyond translation and bilingual coaches.
- It is unclear whether milk is really a good alternative. Babies develop "tooth rot" when they drink sugary milk in their bottle overnight.
- The integration of the intervention into dental care could be more robust (e.g., provide feedback to the dentists that refer their clients so that dentists could support/confirm study messages given to patients).
- While the examination of the outcome, "time to relapse", may be novel, it is unclear if this variable is linked to poor outcomes (e.g., is this outcome appropriate and clinically meaningful). the use of this outcome (versus just early childhood caries by itself) lacks adequate citation and justification.
- Given the multicomponent approach, it may be challenging to determine if it was the water delivery itself or the counselling or both that leads to changes in the primary outcome.
- It does not appear that good measures will be collected to support the cost-effectiveness of both counseling by dietitians and water distribution, in order to justify a larger effectiveness trial.
- The number of mediators and moderators hypothesized seems large for the sample size, bringing the possibility of finding an association by chance.
- Inclusion of children under two years of age might be problematic given their inability to drink out of the bottle provided (Perhaps restrict enrollment to those children who can hold a cup and drink water out of the bottle provided).

**5. Environment:**
Strengths

- The resources are appropriate for conducting the research.

Weaknesses

- The majority of the letters of support are dated from the original submission and will be two years old by the time of the study (it would be nice to have evidence these letters are still active, especially related to cost of the water bottles).

**Clinical Trial Documentation:** *[Not Addressed by Reviewer]*

**Plans for Patient Recruitment/Retention:** *[Not Addressed by Reviewer]*

**Safety Monitoring:** *[Not Addressed by Reviewer]*

**Protections for Human Subjects:** Acceptable Risks and/or Adequate Protections
Data and Safety Monitoring Plan: Acceptable

**Inclusion of Women, Minorities and Children:**

- Sex/Gender: Distribution justified scientifically
- Race/Ethnicity: Distribution justified scientifically
- For NIH-Defined Phase III trials, Plans for valid design and analysis: Not applicable
- Inclusion/Exclusion of Children under 18: Including ages <18; justified scientifically

**Vertebrate Animals:** Not Applicable (No Vertebrate Animals)

**Biohazards:** Not Applicable (No Biohazards)

**Resubmission:** The application addresses many of the comments of the prior reviewers in the "Introduction to the Application" and throughout the grant. However, one point of particular interest was not addressed from the initial submission: there is no plan included detailing what happens with the counseling or home delivery if participants become bored or worn-out discussing solely sugary drinks during the length of the intervention period. Thus it remains unclear if an alternative drink delivery might be considered (such as noncaloric flavored water), a pared-down intervention, or alternative behavioral targets. This comment is particularly meaningful for the adults in the home, who may want "diet" beverages instead of water.

**Resource Sharing Plans:** Acceptable

**Authentication of Key Biological and/or Chemical Resources:** Not Applicable (No Relevant Resources)

**Budget and Period of Support:** Recommend as Requested

- Budgeting for one dietitian to counsel all intervention families over one year (with multiple contacts) may not be enough.

**Advice to Applicants:** Given the multicomponent approach, it may be challenging to determine if it was the water delivery itself or the counselling or both that leads to changes in the primary outcome. It is unclear if there has been any thought to four arms: usual care, water only, water + counseling, counseling only. It just seems unlikely that counseling by dietitians and water distribution could occur cost-effectively in a larger, effectiveness trial (and the investigators would want good evidence to support the cost of both).

**CRITIQUE 4**

| | |
|---|---|
| Significance: | 2 |
| Investigator(s): | 1 |
| Innovation: | 2 |
| Approach: | 2 |
| Environment: | 1 |

**Overall Impact:** The goal of the project is to assess a dietary intervention, replacing sugar sweetened beverages (SSBs) with water, to reduce caries in children with S-ECC, a severe form of early childhood caries. The potential importance of the findings is high, since SSBs have been implicated in ECC and childhood obesity. I have some concern about the effect of ‘drop-in’ diluting the treatment effect. There are a number of programs that aim to reduce SSB consumption.

**1. Significance:**
Strengths

- The investigators are addressing an important problem affecting the health of a sizeable segment of the population.
- The intervention, if successful, could be used to combat more than one health issue.

Weaknesses

- None noted

**2. Investigator(s):**

Strengths

- This is a strong, experienced team with the necessary expertise to carry out the RCT**.**
- There is an established record of collaboration among members of the research team.

Weaknesses

- None noted

**3. Innovation:**

Strengths

- The intervention itself is not all that innovative, but applying this intervention in this population for this indication is innovative.

Weaknesses

- None noted

**4. Approach:**

Strengths

- There are detailed plans for carrying out the study.
- The statistical methods section is strong, with consideration given to handling the interval censored data and departures from model assumptions.
- The sample size calculation describes the power under various assumptions about ‘drop-in’ and compliance.
- Their preliminary data suggest that their retention plan is effective for minimizing study dropout.

Weaknesses

- Although the interval censored analyses are appropriate, it is possible that the width of the follow-up intervals (every six months) might mask treatment differences.
- Adherence is assessed through the collection of water bottles and completion of the BEVQ-15 survey instrument. It is unclear if this is a sufficient way of monitoring adherence.
- The potential for ‘drop-in’, at least in terms of messages to limit the number of SSBs, seems high.

**5. Environment:**

Strengths

- These sites are particularly well suited to carry out these studies since there is a high volume of children at risk for S-ECC.

Weaknesses

- *[Not Addressed by Reviewer]*

**Clinical Trial Documentation:** The study protocol is detailed and comprehensive.

**Plans for Patient Recruitment/Retention:** Appropriate, with ready access to the intended patient population and a retention plan that they have used successfully in the past.

**Safety Monitoring:** Appropriate and detailed.

**Protections for Human Subjects:** Acceptable Risks and/or Adequate Protections

Data and Safety Monitoring Plan: Acceptable

**Inclusion of Women, Minorities and Children:**
- Sex/Gender: Distribution justified scientifically
- Race/Ethnicity: Distribution justified scientifically
- For NIH-Defined Phase III trials, Plans for valid design and analysis: Scientifically acceptable
- Inclusion/Exclusion of Children under 18: Including ages <18; justified scientifically
- It's a study in a pediatric population.

**Vertebrate Animals:** Not Applicable (No Vertebrate Animals)

**Biohazards:** Not Applicable (No Biohazards)

**Resubmission:** The investigators were responsive to comments on the previous application.

**Resource Sharing Plans:** Acceptable

**Authentication of Key Biological and/or Chemical Resources:** Not Applicable (No Relevant Resources)

**Budget and Period of Support:** Recommend as Requested

**CRITIQUE 5**

| | |
|---|---|
| Significance: | 2 |
| Investigator(s): | 1 |
| Innovation: | 3 |
| Approach: | 4 |
| Environment: | 1 |

**Overall Impact:** This application seeks to enroll 260 children aged zero through eight into a two-arm randomized study investigating the potential impact on dental caries of a home-environment intervention that facilitates and emphasizes water consumption over sugar-sweetened beverages (SSBs) in a population at high risk for dental caries, where all participants will receive broader nutrition education and where the active intervention group will additionally receive family-focused counseling and free deliveries of fluoridated water in appealing animal-shaped bottles. Although there would still be a need for follow-up studies in the event that the intervention has an impact on dental caries to investigate how to scale up the intervention in a realistic way, information on the efficacy of the intervention in a high-risk population would have considerable value from a public-health perspective.

**1. Significance:**
Strengths
- Early childhood caries has substantial and long-lasting impacts.
- Factors associated with early childhood caries are also associated with obesity, linking these two domains of health in ways where effective treatments for one condition can be expected to be helpful for the other.

Weaknesses
- There would still be a need for follow-up research on developing sustainable variations on the active intervention in the event that the intervention proves to have a substantial impact.

**2. Investigator(s):**
Strengths

- Investigative team has strengths in dentistry, public health, epidemiology, and biostatistics.

Weaknesses

- *[Not Addressed by Reviewer]*

**3. Innovation:**

Strengths

- Packaging fluoridated water in bottles that have already in effect been test-marketed by zoos is innovative.
- Analogy of providing healthy beverages to providing safe drinking water in areas where water supplies are unsafe is innovative.
- “Thirsty for a Smile” is a clever tag-line for the project.

Weaknesses

- *[Not Addressed by Reviewer]*

**4. Approach:**

Strengths

- Multi-disciplinary collaboration with Yakima Valley Farm Workers Clinic provides a helpful foundation for the proposed study.
- Power calculations appropriately assume smaller effect sizes than those associated with point estimates from previous research.

Weaknesses

- Translation of findings is apt to remain a concern given the realistic scenario that distribution of water will not be free for the greater part of the population.
- Adjustment for time-dependent covariates has the potential to adjust away part of an intervention effect.
- Plans for causal mediation analysis should be supplemented with sensitivity analyses given knowledge about how sensitive such analyses can be to underlying assumptions.

**5. Environment:**

Strengths

- The environment for carrying out the proposed research is excellent.

Weaknesses

- *[Not Addressed by Reviewer]*

**Clinical Trial Documentation:** Application includes appropriate documentation.

**Plans for Patient Recruitment/Retention:** Application includes plans for patient recruitment/retention.

**Safety Monitoring:** Application includes documentation of plans for monitoring data accuracy and maintaining data privacy.

**Protections for Human Subjects:** Acceptable Risks and/or Adequate Protections

- Application documents protections for the minimal risks described in the application.

Data and Safety Monitoring Plan: Acceptable

- Application describes adequate procedures for data monitoring and security.

**Inclusion of Women, Minorities and Children:**

- Sex/Gender: Distribution justified scientifically
- Race/Ethnicity: Distribution justified scientifically
- For NIH-Defined Phase III trials, Plans for valid design and analysis: Not applicable
- Inclusion/Exclusion of Children under 18: Including ages <18; justified scientifically

- Although project title focuses specifically on Latino children, the project will appropriately enroll families without reference to gender, race, and ethnicity, with an expectation that roughly half of the sample will be Latino. Inclusion of children under age 18 is justified by their being at heightened risk for dental caries.

**Vertebrate Animals:** Not Applicable (No Vertebrate Animals)

**Biohazards:** Not Applicable (No Biohazards)

**Resubmission:** Introduction to revised application addresses previous critiques.

**Resource Sharing Plans:** Acceptable

**Authentication of Key Biological and/or Chemical Resources:** Not Applicable (No Relevant Resources)

**Budget and Period of Support:** Recommend as Requested

**THE FOLLOWING SECTIONS WERE PREPARED BY THE SCIENTIFIC REVIEW OFFICER TO SUMMARIZE THE OUTCOME OF DISCUSSIONS OF THE REVIEW COMMITTEE, OR REVIEWERS' WRITTEN CRITIQUES, ON THE FOLLOWING ISSUES:**

**PROTECTION OF HUMAN SUBJECTS: ACCEPTABLE**

**INCLUSION OF WOMEN PLAN: ACCEPTABLE**

**INCLUSION OF MINORITIES PLAN: ACCEPTABLE**

**INCLUSION OF CHILDREN PLAN: ACCEPTABLE**

**COMMITTEE BUDGET RECOMMENDATIONS: The budget was recommended as requested.**

---

Footnotes for 1 U01 DE027026-01A1; PI Name: Cunha-Cruz, Joana

NIH has modified its policy regarding the receipt of resubmissions (amended applications). See Guide Notice NOT-OD-14-074 at http://grants.nih.gov/grants/guide/notice-files/NOT-OD-14-074.html. The impact/priority score is calculated after discussion of an application by averaging the overall scores (1-9) given by all voting reviewers on the committee and multiplying by 10. The criterion scores are submitted prior to the meeting by the individual reviewers assigned to an application, and are not discussed specifically at the review meeting or calculated into the overall impact score. Some applications also receive a percentile ranking. For details on the review process, see http://grants.nih.gov/grants/peer_review_process.htm#scoring.
